# Editorial: Frontiers’ Research Topic “Cancer Vaccines: Time to Think Differently!”

**DOI:** 10.3389/fimmu.2021.771319

**Published:** 2021-10-15

**Authors:** Stephanie E. B. McArdle, Graham Pawelec, Alan Graham Pockley, Pål Johansen

**Affiliations:** ^1^ John Van Geest Cancer Research Centre, Nottingham Trent University, Nottingham, United Kingdom; ^2^ Centre for Health, Ageing and Understanding Disease (CHAUD), School of Science and Technology, Nottingham Trent University, Nottingham, United Kingdom; ^3^ Department of Immunology, Institute for Cell Biology, University of Tuebingen, Tuebingen, Germany; ^4^ Cancer Solutions Program, Health Sciences North Research Institute (HSNRI), Sudbury, ON, Canada; ^5^ Department of Dermatology, University Hospital Zurich, Zurich, Switzerland

**Keywords:** combined immunotherapies, cancer vaccine combination, novel approach, dendritic cells, PD1 and PDL1

Although the advent of checkpoint inhibitors has revolutionised immunotherapy, the surge of optimism has been quickly dampened by the fact that only some cancers and only a proportion of cancer patients truly benefit from these treatments when they are administered as a monotherapy. The era of combined therapy is now upon us and many clinical trials are now combining drugs, vaccines and checkpoint inhibitors with the aim to amplify the ability of the immune system to recognize and eradicate cancer. This Frontiers Research Topic entitled “Cancer vaccines: Time to think differently!” has collated 16 contributions from experts who are exploring a range of novel treatment approaches that are centred on vaccine-based approaches for triggering protective anti-cancer immunity in pre-clinical animal models and patients.

In a mouse model of head and neck squamous cell carcinoma, Jin et al. show that combining radiation and cetuximab (an epidermal growth factor receptor inhibitor) increased intra-tumoral infiltration of NK cells and CD8^+^ T cell and enhanced the expression of PD-L1 (checkpoint pathway ligand) by tumour cells. As a consequence, this heightened the susceptibility of the tumour to PD-L1 antibody treatment and thereby increased durable tumour regression and the survival of mice when all three treatments were combined. However, this is only one example of many new and different strategies that are currently being investigated, as has been highlighted in the review article by Chen et al. Although the current focus is very much on ‘checkpoint blockade’, there is no doubt that future combination strategies are likely to include metabolic and epigenetic therapies to circumvent immune escape mechanisms and block intricate immunosuppressive mechanisms in the tumour microenvironment (TME), as discussed by Chen et al. or the use of *in situ* ablation, as discussed by van den Bijgaart et al.

The successful application of platforms employing mRNA-based cancer vaccine technology to SARS-CoV-2 vaccines and the remarkable success of these mRNA-based formulations against COVID-19 has once again highlighted the potency of this novel approach in cancer. This success has reawakened awareness of the potential potency of earlier approaches to vaccines such as the use of dendritic cells (DCs), as shown by Kumbhari et al. who has used a theoretical approach which applies a mathematical model and simulations to demonstrate how vaccine-induced avidity selection can influence tumour clearance. They showed that treatment with immature DCs has the potential to promote the selective expansion of high-avidity cytotoxic T lymphocytes (CTLs) and lead to tumour regression. “Classical” approaches to the generation of DCs for use in immunotherapy may also still leave room for improvement, as discussed by Calmeiro et al. Alternatively, using delivery systems such as the DNA-based ImmunoBody^®^ which directly targets immature DCs *in vivo* offers the opportunity to simultaneously trigger immunity to two antigens, HAGE and WT1, as described by Almshayakhchi et al. Although predicted epitopes derived from vaccines can be used to monitor CD8^+^ T cell responses *ex vivo* in clinical trial settings as a means to gauge the success of T cell vaccines, Lehmann et al. showed that there was no correlation between the ranking of epitopes on the prediction scale and their actual immune dominance. One would therefore need to screen large vaccine-derived peptide pools to increase the accuracy of the targeted response.

Data from murine models presented by Bikorimana et al. demonstrate that thymoproteasome-based proteasomal alterations can trigger potent T cell immunity when used as part of an engineered mesenchymal stromal cell-based vaccine. Although vaccination led to the recruitment of macrophages and DCs, the immunotherapeutic effect was mediated by cross-priming-dependent DCs. It was also noted that an interaction between vaccine and monocytes/macrophages impaired T cell activation, as a consequence of which the depletion of monocytes/macrophages prior to vaccination increased efficacy. In an additional article on the use of DC-based vaccines, Stevens et al. review nearly 20 years of DC-based immunotherapy in lung cancer. They conclude that combining DC-based immunotherapy with other cancer therapies, such as chemotherapy, radiotherapy and/or checkpoint inhibition may potentially improve vaccine efficacy. Clinical studies testing these hypotheses are underway.

Alternative new and previously considered cancer vaccine delivery approaches are also being (re)-evaluated and discussed in the field. Oladejo et al. consider *Listeria monocytogenes* as a vaccine vector. They discuss recent clinical experience with Listeria-based immunotherapies and recent advances in the development of improved Listeria-based vaccine platforms and their utilization. In an elegant approach by Otterhaug et al., a subunit cancer vaccine is combined with a photochemical compound in a so-called photochemical internalisation (PCI). The intradermal administration of the vaccine is followed by its uptake into skin antigen presenting cells (APCs). Subsequent light treatment disrupts vaccine-containing endosomes and triggers the release of antigen to the cytosol for presentation to major histocompatibility (MHC) class I molecules and stimulation of CD8^+^ T cell responses. This first-in-human phase I study in healthy volunteers assessed safety, tolerability, and immune responses to PCI vaccination in combination with the adjuvant poly-ICLC. Another approach to cancer vaccination is presented by Zhang et al. who describe a personalized vaccination regime that could be applied for both the therapeutic and prophylactic treatment of lung cancer. This is based on the derivation of lung cancer cells from induced pluripotent stem cells (iPSCs), which are modified to express Cre-dependent tumour antigens. Subsequent viral delivery (e.g. *via* Adenovirus) of Cre activated exogenous driver mutations and resulted in the transformation of lung cancer cells. This “Virus-Infected Reprogrammed Somatic cell-derived Tumour cell vaccination” (VIReST), primed tumour-specific T cell responses that significantly prolonged survival in mouse models of lung cancer.

Finally, a vaccine cannot be better than its adjuvant. Especially in the context of cancer vaccines, the strength and quality of adjuvants is essential if one is to overcome the self-tolerance barrier of barely immunogenic tumour antigens. As discussed by Cuzzubo et al., the use of carefully selected adjuvants to improve vaccine potency in older patients becomes crucial and although some cancers can be completely protected against by vaccinating early in life, as discussed by Crews et al., the majority will rely on the use of adjuvants capable of inducing efficient and long lasting T_RM_ cells. These may have a progenitor exhausted phenotype such as the one described by León-Letelier et al., which have been shown to control the disease and lead to better responses to PD-1 immunotherapy. Adjuvants may also offer a means to overcome the immune suppressive TME, as discussed by Paston et al.

Cancer develops over many years during which time cells have accumulated numerous genetic alterations and been continuously “sculpted”/modified by the immune system. This leads to the emergence of one fully malignant escapee which then goes on to form a tumour having an immunosuppressive TME. However, although one fully malignant cell represents the origin of tumour growth and spread, many other cells at different stages of potential disease progression remain even if the tumour has been cured. Hence, in addition to the challenging task of eliminating the primary tumour to prevent metastasis and relapse, future treatments will have to take this into consideration. There is no longer any doubt that our lifestyle and age will affect our immune system and the entirety of the soma, all of which will influence our ability to respond to novel combinatorial treatments such as those detailed in this Research Topic. It is our firm belief that we will not be able to cure/eradicate cancer unless we are able to harness ways to implement a more holistic approach to cancer treatments (and cancer prevention in the first place).

## Author Contributions

All authors listed have made a substantial, direct, and intellectual contribution to the work and approved it for publication.

## Conflict of Interest

The authors declare that the research was conducted in the absence of any commercial or financial relationships that could be construed as a potential conflict of interest.

## Publisher’s Note

All claims expressed in this article are solely those of the authors and do not necessarily represent those of their affiliated organizations, or those of the publisher, the editors and the reviewers. Any product that may be evaluated in this article, or claim that may be made by its manufacturer, is not guaranteed or endorsed by the publisher.

